# COMFFETI, Combined Fresh and Frozen Embryo Transfers per Individual: A New Index of Quality Control for The Performance of Embryologic Labs in The Emerging Era of Segmentation of Cycle and Freeze-All Strategy

**DOI:** 10.22074/ijfs.2019.5424

**Published:** 2018-10-02

**Authors:** Papanikolaou Evangelos G., Evi Timotheou, Petroula Tatsi, Hieronymus Janssens, Michael Grynberg, Apostolos Athanasiadis, Christina Zafeirati, Robert Najdecki, Stamatios Petousis

**Affiliations:** 1 Assisting NATURE, Unit of Human Reproduction and Genetics, Thessaloniki, Greece; 2 University Hospital of Brussels, Dutch-speaking Free University of Brussels, Brussels, Belgium; 3 Aristotle University of Thessaloniki, 3rd Department Ob Gyn, Thessaloniki, Greece; 4 Department of Reproductive Medicine, Hôpital Jean Verdier, Avenue du 14 Juillet, Bondy, France

**Keywords:** Embryo, Fresh, Frozen, Infertility

## Abstract

The efficacy of *in vitro* fertilization (IVF) for treating human infertility has only one final efficacy index and that is the achievement of a delivery. However, with the evolution of the freeze-all strategy, a new problem is arising for evaluating the performance of an embryological team. The aim of the study was to present a new representative index, combining fresh and frozen embryo transfer success rates. In this opinion article, apart from the effectiveness of managing fresh gametes and embryos, we wish to evaluate the efficacy of the processes of both freezing and thawing of the produced embryos. The reporting of pregnancy rates of an IVF unit in the past was primarily laying in the fresh embryo transfer (ET) pregnancy rates. Now with the most frequent utilization of freeze-all strategy, it does not seem logical to report only on poor prognosis patients as all the good cases are postponed for thawed cycles. Ongoing implementation of the freeze-all strategy has indicated the need to establish a new representative index that may combine the success of both fresh and frozen cycles performed in the same woman; an index that may not be biased by the policy of an IVF center towards or against the freeze-all strategy. This newly proposed index, which is referred to as COMFFETI (Combined Fresh & Frozen Embryo Transfers per Individual), describes the optimal way to report final reproductive outcomes in the present opinion article.

Currently, the efficacy of *in vitro* fertilization (IVF) for treating human infertility has only one final efficacy index, which is reaching a successful delivery. However, there are so many confounding factors that might alter the achievement of a pregnancy. These factors are either presently unmanageable by medicine, such as the genetic quality of the produced eggs, sperms and the endometrial receptivity capacity of each individual, or manageable by current technology, such as gonadotrophin capacity, equipment capacity, culture media capacity, air quality, scientist’s expertise and so on (1, 2). In the last category the embryological team, who use the abovementioned techniques and equipment on a daily basis, play the central role for an optimal outcome (3).

Although, there are certain laboratory parameters that can be utilized to monitor the efficacy of a laboratory team, such as fertilization rate, degeneration rate, cleavage rate, blastulation rate, proportion of embryos for freezing, unfortunately the embryological staff are eventually solely judged by the pregnancy rates that their lab is achieving. This is unfortunate, because many confounders that intercede after their last involvement, such as the type of embryo transfer catheter, the capacity of the medical transferee, the quality of the endometrium, or the quality of the luteal support, are factors that can be critical for the efficiency of their work (4, 5). Moreover, with the increased number of freezing and sequential thawing steps, the vitrification technique is also considered as a new confounder in the evaluation of the embryologists’ performance.

With the evolution of the Freeze-all strategy, a new problem is arising for evaluating the performance of an embryological team. Apart from the efficacy of the management of fresh gametes and embryos, we shall also take into consideration the efficacy of both freezing and thawing embryos. These are two extra procedures, which are both dependent on the expertise of the person who is performing these steps, therefore might potentially be at risk for mistakes. Moreover, the cryopreservation of all high responders and presumably good prognosis patients complicate the situation even more, as only poor or average responders are allowed to proceed with embryo transfer (ET), putting the probability of pregnancy with fresh embryo at risk in these poor prognosis patients.

Previously, the reports on pregnancy rates of an IVF unit were primarily based on the cases with fresh ET. Now with the frequent utilization of the freeze-all strategy there is a risks of reporting only poor prognosis patients, as all the good cases are postponed for thawed cycles.

The rationale for the freeze-all strategy and eventually segmentation of the IVF cycle has developed over the recent years and is based on two pathophysiological facts. The main one is the endometrial receptivity, which is definitely violated during ovarian stimulation due to the supra-physiological levels of the steroid hormones. Devroey et al. (6) have clearly demonstrated with endometrial biopsies in both agonist and antagonist protocols, that the early luteal endometrial histology is always advanced. Moreover, when this advancement lasts for more than 3 days, the endometrium may be considered as out-of-phase, which in turn significantly reduces the possibility of pregnancy. The second reason is the complete eradication of severe ovarian hyperstimulation syndrome (OHSS) when fresh ET is withheld. Then, starting the next month, ET can be performed in a more naturally prepared endometrium with the absence of any risks for OHSS (7).

Ongoing implementation of the freeze-all strategy has indicated the need to establish a new representative index that may combine the success of both fresh and frozen cycles administered in the same woman, an index that may not be biased by the policy of an IVF center in favor of or against the freeze-all strategy (8, 9). This new index proposed as COMFFETI (Combined Fresh & Frozen Embryo Transfers per Individual) is described in the present opinion article.

The proposed COMFFETI index could be defined as a binomial variable [yes (1) or no (0)] reflecting the achievement of a pregnancy or not per individual (couple) at the end of each stimulated cycle; including fresh ET plus the thawed ET obtained by a single multifollicular ovarian stimulation cycle. This is a radically different index from the widely used index of pregnancy/delivery rate per transfer.

The basic difference lies in the fact that by the old way of reporting clinical or ongoing pregnancy per fresh ET reflects the potential of pregnancy achievement following this specific fresh ET (8), without considering the additional potential of success of surplus embryos obtained after the same ovarian stimulation. Therefore, an individual that may finally achieve a pregnancy at her third frozen ET (1 fresh and two frozen cycles failed) is considered as having a 0% pregnancy rate in her first attempt, while if the COMFFETI index would have been applied, this woman would have a 100% COMFFETI index, as she would have achieved a pregnancy with embryos still from the initial ovarian stimulation, even at her third frozen embryo transfer.

**Table 1:** Ηypothetical outcomes for hypothetical couples and potential reports in clinic A, which is fresh-cycle friendly


Patient	Fresh COCs	Produced embryos	Destiny	ET (n)	Cryo (n)	Fresh outcome	1^s^^t^ frozen	2^n^^d^ frozen	DR/ET	COMFFETI

1	2	1D2	Fresh ET	1D2	0	Neg	-	-		
2	9	6D3	Fresh ET	2D3	4D3	Delivery	-	-		
3	17	6D5	Fresh ET	2D5	4D5	Delivery	-	-		
4	12	5D5	Fresh ET	1D5	4D5	Neg	Neg	Neg		
5	15	4D5	Fresh ET	1D5	3D5	Neg	Neg	Delivery		
6	9	2D3	Fresh ET	2D3	2D3	Neg	-	-		
7	11	6D3	Fresh ET	2D3	4D3	Neg	Neg	Neg		
8	5	2D3	Fresh ET	2D3	0	Neg	-	-		
9	10	5D5	Fresh ET	2D5	3D5	Delivery	-	-		
10	10	6D3	Fresh ET	2D3	4D3	Neg	Neg	Neg		
Overall									30% (3/10)	40% (4/10)


COCs; Cumulus oocytes, ET; Embryo transfer, Neg; Negative, and DR/ET; Delivery rate per fresh embryo transfer.

**Table 2:** Hypothetical outcomes for the same hypothetical couples and potential reports in clinic B, which is freeze-ALL-friendly


Patient	Fresh COCs	Produced embryos	Destiny	ET (n)	Cryo (n)	Fresh outcome	1^s^^t^ frozen	2^n^^d^ frozen	DR/ET	COMFFETI

1	2	1D2	Fresh ET	1D2	0	Neg	-	-		
2	9	6D3	Fresh ET	2D3	4D3	Delivery	-	-		
3	17	6D5	FRALL	0	6D5	No ET	Delivery	-		
4	12	5D5	Fresh ET 1D5	1D5	4D5	Neg	Neg	Neg		
5	15	4D5	FRALL	0	4D5	No ET	Neg	Delivery		
6	9	2D3	FRALL	0	2D3	No ET	Delivery	-		
7	11	6D3	Fresh ET 2D3	2D3	4D3	Neg	Neg	Neg		
8	5	2D3	Fresh ET 2D3	2D3	0	Neg	-	-		
9	10	5D5	FRALL	0	5D5	No ET	Delivery	-		
10	10	6D3	FRALL	0	6D3	No ET	Delivery	-		
Overall									20% (1/5)	60% (6/10)


COCs; Cumulus oocytes, FRALL; Freeze all, ET; Embryo transfer, Neg; Negative, and DR/ET; Delivery rate per fresh embryotransfer.

The value of this new index could be demonstrated in case we examine two theoretical examples of different IVF centers, of which the first is in favor of fresh ET, while the second is practicing the freeze-all technique. Table 1 presents the final outcomes of 10 patients undergoing IVF treatment in an IVF center performing fresh ET. In case there were three deliveries achieved by the first ET with fresh embryos, the traditional index would have been 30%. Table 2 presents the same 10 patients undergoing IVF treatment and their final outcomes in a center taking the freeze-all approach. In case an IVF Unit has as mainstream policy to freeze embryos, the traditional delivery index would be only 20%. For instance case number 5 (due to high response) and case number 10 (due to high follicular progesterone) withheld the fresh transfer and took the freeze-all approach. Therefore, a superficial outcome of the two centers would indicate that centers favoring the fresh ET policy are more successful in achieving clinical pregnancies/deliveries.

Our own data support what was mentioned in the examples above about the COMFETI index. Based on the latest 50 cases of fresh embryo transfer cycles, the live birth rate has been 46% for the first embryo transfer, while the COMFFETI index has been 74% after two embryo transfers. Relatively, for the last 50 cases of the freeze-all strategy, the live birth rate was 58% for the first embryo transfer per case while the COMFFETI index was 82%. These results indicate the importance of using an index, which reflects the cumulative results of consecutive embryo transfers, especially in the freeze-all-friendly centers.

However, the consideration of pregnancies achieved “at the end of the day” according to COMFFETI index would radically change the situation. COMFFETI index would only have a slight increase from 20 to 30% in the first center, favoring fresh ETs, while in the second example, both cases 5 and 10 might have achieved a delivery with frozen embryos, and thus COMFFETI pregnancy rate could rise up to 60%, representing a totally different clinical outcome with regards to traditional index.

The additional great advantage that COMFFETI pregnancy rate provides is that it incorporates the implantation potential of all embryos produced from a single stimulated cycle. On the other hand, a drawback might be that COMFFETI rate is significantly related to the efficacy of cryopreservation techniques and the assumed increased success rates reported from the frozen cycles (10). Therefore, a complete freeze-all-friendly center may not actually achieve a pregnancy from the very first fresh embryo transfer with the traditional pregnancy rates being even 0%. Furthermore, one of the shortcomings of COMFFETI index is that you cannot include all the couples’ result into assessment, since some couples may take a long time to transfer all their vitrified embryos for ET. However, given the fact that especially when blastocysts are cryopreserved and subsequently all of them are transferred in consecutive natural cycles with assumed receptive endometria, an increased absolute number of clinical pregnancies might actually be achieved by this strategy (11).

The basic endpoint of a successful IVF treatment cycle is giving a healthy baby to the mother (12). The interval required for such a purpose may not be the primary concern of a rationale patient. The main concern that preoccupies every subfertile woman or man is the probability of having a healthy baby after a stimulated cycle. It is mentally much more encouraging to tell the woman or the couple that there is a 60% cumulative chance of a successful pregnancy by transferring all the embryos obtained through a single ovarian stimulation, rather than a 20% chance from the first fresh ET.

The COMFFETI index in reproductive medical practice may be used to give the infertile patients a more subjective view about the realistic possibilities to have a successful IVF cycle from the beginning of the treatment. Moreover, reporting to organizations like European Society of Human Reproduction and Embryology (ESHRE) and Center for Disease Control would become increastingly objective by using the above index as a higher number of centers are moving towards the freeze-all policy, and therefore only poor cases or modest responders would be selected for fresh embryo transfers (13, 14).
